# Deriving Normative Data on 24-Hour Ambulatory Blood Pressure Monitoring for South Asian Children (ASHA): A Clinical Research Protocol

**DOI:** 10.1177/20543581211072329

**Published:** 2022-01-31

**Authors:** Samina Nazarali, Cal H. Robinson, Farah Khan, Tayler Pocsai, Dipika Desai, Russell J. De Souza, Girish Bhatt, Allison Dart, Janis Dionne, Salma Elmansy, Sujane Kandasamy, Scott A. Lear, Joyce Obeid, Rulan Parekh, Zubin Punthakee, Rajiv Sinha, Lehana Thabane, Gita Wahi, Michael Zappitelli, Sonia S. Anand, Rahul Chanchlani

**Affiliations:** 1 Michael G. DeGroote School of Medicine, McMaster University, Hamilton, ON, Canada; 2 Division of Nephrology, Department of Paediatrics, The Hospital for Sick Children, Toronto, ON, Canada; 3 Population Health Research Institute, Hamilton, ON, Canada; 4 Department of Health Research Methods, Evidence and Impact, McMaster University, Hamilton, ON, Canada; 5 Department of Pediatrics, All India Institute of Medical Sciences, Bhopal, India; 6 Division of Nephrology, Department of Pediatrics, University of Manitoba, Winnipeg, Canada; 7 Division of Nephrology, Department of Pediatrics, BC Children’s Hospital, The University of British Columbia, Vancouver, Canada; 8 Department of Pediatrics, McMaster University, Hamilton, ON, Canada; 9 Simon Fraser University, Burnaby, BC, Canada; 10 Centre for Metabolism, Obesity and Diabetes Research, McMaster University, Hamilton, ON, Canada; 11 Pediatric Nephrology, Department of Pediatrics, Institute of Child Health, Kolkata, India; 12 Department of Medicine, McMaster University, Hamilton, ON, Canada; 13 Division of Pediatric Nephrology, Department of Pediatrics, McMaster University, Hamilton ON, Canada; 14 ICES McMaster, McMaster University, Hamilton, ON, Canada

**Keywords:** hypertension, blood pressure, ambulatory blood pressure monitoring, South Asian, pediatric, children

## Abstract

**Background:**

The global prevalence of hypertension in children and adolescents has increased over the past 2 decades and is the strongest predictor of adult hypertension. South Asians have an increased prevalence of metabolic syndrome associated risk factors including abdominal obesity, diabetes, and hypertension. All these factors contribute to their increased cardiovascular disease burden. Accurate and early identification of hypertension in South Asian children is a necessary aspect of cardiovascular disease prevention. Ambulatory blood pressure monitoring (ABPM) is considered the gold-standard for pediatric blood pressure (BP) measurement. However, its utilization is limited due to the lack of validated normative reference data in diverse, multiethnic pediatric populations.

**Objective:**

The primary objective is to establish normative height-sex and age-sex-specific reference values for 24-h ABPM measurements among South Asian children and adolescents (aged 5-17 years) in Ontario and British Columbia, Canada. Secondary objectives are to evaluate differences in ABPM measurements by body mass index classification, to compare our normative data against pre-existing data from German and Hong Kong cohorts, and to evaluate relationships between habitual movement behaviors, diet quality, and ABPM measurements.

**Design:**

Cross-sectional study, quasi-representative sample.

**Setting:**

Participants will be recruited from schools, community centers, and places of worship in Southern Ontario (Greater Toronto and Hamilton area, including the Peel Region) and Greater Vancouver, British Columbia.

**Participants:**

We aim to recruit 2113 nonoverweight children (aged 5-17 years) for the primary objective. We aim to recruit an additional 633 overweight or obese children to address the secondary objectives.

**Measurements:**

Ambulatory BP monitoring measurements will be obtained using Spacelabs 90217 ABPM devices, which are validated for pediatric use. The ActiGraph GT3X-BT accelerometer, which has also been validated for pediatric use, will be used to obtain movement behavior data.

**Methods:**

Following recruitment, eligible children will be fitted with 24-h ABPM and physical activity monitors. Body anthropometrics and questionnaire data regarding medical and family history, medications, diet, physical activity, and substance use will be collected. Ambulatory BP monitoring data will be used to develop height-sex- and age-sex-specific normative reference values for South Asian children. Secondary objectives include evaluating differences in ABPM measures between normal weight, overweight and obese children; and comparing our South Asian ABPM data to existing German and Hong Kong data. We will also use compositional data analysis to evaluate associations between a child’s habitual movement behaviors and ABPM measures.

**Limitations:**

Bloodwork will not be performed to facilitate recruitment. A non-South Asian comparator cohort will not be included due to feasibility concerns. Using a convenience sampling approach introduces the potential for selection bias.

**Conclusions:**

Ambulatory BP monitoring is a valuable tool for the identification and follow-up of pediatric hypertension and overcomes many of the limitations of office-based BP measurement. The development of normative ABPM data specific to South Asian children will increase the accuracy of BP measurement and hypertension identification in this at-risk population, providing an additional strategy for primary prevention of cardiovascular disease.

## Abrégé

**Contexte:**

La prévalence mondiale de l’hypertension chez les enfants et les adolescents a augmenté au cours des deux dernières décennies et constitue le plus important facteur prédictif de l’hypertension chez les adultes. Le syndrome métabolique associé aux facteurs de risque que sont l’obésité abdominale, le diabète et l’hypertension est plus prévalent chez les personnes d’origine sud-asiatique. Tous ces facteurs contribuent à une charge de morbidité cardiovasculaire accrue pour ces personnes. Le dépistage précis et précoce de l’hypertension chez les enfants d’Asie du Sud est un aspect incontournable de la prévention des maladies cardiovasculaires. Le monitoring ambulatoire de la pression artérielle (MAPA) est considéré comme la norme pour la mesure de la pression artérielle chez les enfants. Son utilization est toutefois limitée en raison de l’absence de références normatives validées dans des populations pédiatriques diversifiées et multiethniques.

**Objectifs:**

L’objectif principal est d’établir des valeurs de référence normatives taille-sexe et âge-sexe pour les mesures de MAPA sur 24 heures chez les enfants et les adolescents d’origine sud-asiatique (âgés de 5-17 ans) de l’Ontario et de Colombie-Britannique (Canada). Les objectifs secondaires sont : 1) d’évaluer les différences dans les mesures de MAPA selon une classification basée sur l’indice de masse corporelle; 2) de comparer nos données normatives aux données préexistantes tirées de cohortes d’Allemagne et de Hong Kong, et 3) d’évaluer les relations entre les comportements actifs habituels, la qualité de l’alimentation et les mesures de MAPA.

**Type d’étude:**

Étude transversale avec échantillon quasi représentatif.

**Cadre:**

Les participants seront recrutés dans des écoles, des centers communautaires et des lieux de culte du sud de l’Ontario (région du Grand Toronto et de Hamilton, y compris la région de Peel) et du Grand Vancouver en Colombie-Britannique.

**Sujets:**

Nous souhaitons recruter 2113 enfants (5 à 17 ans) ne présentant pas de surpoids pour l’objectif principal. Et 633 enfants en surpoids ou obèses pour les objectifs secondaires.

**Mesures:**

Les mesures de MAPA seront obtenues à l’aide d’appareils Spacelabs 90217 validés pour un usage pédiatrique. L’accéléromètre ActiGraph GT3X-BT, également validé pour un usage pédiatrique, sera utilisé pour colliger des données sur le comportement actif.

**Méthodologie:**

Après le recrutement, les enfants admissibles seront équipés d’un appareil de MAPA pour 24 heures et de moniteurs d’activité physique. Les caractéristiques anthropométriques et les données d’un questionnaire portant sur les antécédents médicaux et familiaux, la médication, l’alimentation, l’activité physique et la consommation de substances seront recueillies. Les données de MAPA seront utilisées pour établir des valeurs de référence normatives taille-sexe et âge-sexe pour les enfants d’Asie du Sud. Les objectifs secondaires comprennent l’évaluation des différences dans les mesures de MAPA selon que les enfants ont un poids normal, un surpoids ou sont obèses, et la comparaison de nos données de MAPA pour des enfants d’Asie du Sud avec les données existantes en Allemagne et à Hong Kong. Nous procèderons également à l’analyze de composition des données afin d’évaluer les relations entre les comportements actifs habituels de l’enfant et les mesures de MAPA.

**Limites:**

Pour faciliter le recrutement, les analyses sanguines ne seront pas effectuées. Aucune cohorte de comparaison constituée de sujets non originaires d’Asie du Sud ne sera incluse en raison de problèmes de faisabilité. L’emploi d’une approche d’échantillonnage de commodité introduit un possible biais de sélection.

**Conclusion:**

Le MAPA est un outil précieux pour le dépistage et le suivi de l’hypertension pédiatrique et elle permet de surmonter plusieurs des limites de la mesure de la PA en cabinet. L’établissement de références normatives de MAPA spécifiques aux enfants d’Asie du Sud permettra d’accroître la précision de la mesure de la PA et le dépistage de l’hypertension dans cette population à risque, fournissant ainsi une stratégie supplémentaire pour la prévention primaire des maladies cardiovasculaires.

## Introduction

Hypertension (HTN) is one of the most common causes of preventable death worldwide^1,2^ and often begins in childhood.^3-6^ The global prevalence of pediatric HTN has increased over the past 2 decades, from 1.3% (1990-1999) to 6.0% (2010-2014),^7^ partly due to the increasing childhood obesity.^8^ Childhood blood pressure (BP) remains the strongest predictor of adult HTN and is associated with an increased risk for adult cardiovascular disease (CVD), including ischemic heart disease and stroke.^3-6^ From a health economics perspective, the costs attributable to HTN in Canada were estimated at $13.9 billion in 2010, rising to $20.5 billion by 2020.^9,10^

Ambulatory blood pressure monitoring (ABPM) is the reference standard for detecting HTN in children and adults.^11-13^ It provides a free-living BP assessment over a 24-h period and mitigates many limitations of office-based BP measurement.^14^ Despite these advantages, pediatric ABPM interpretation is limited by the continued use of normative data derived over 20 years ago from a homogenous white Caucasian population in Germany. Given that BP is known to vary with ethnicity,^15^ this existing normative data may not accurately detect abnormal BP profiles among non-white children. One such population is South Asians (people originating from India, Pakistan, Bangladesh, Nepal and Sri Lanka), which comprise 25% of the world’s population,^16^ and are Canada’s fastest-growing non-white ethnic group.

Alarmingly, emerging data suggests an impending epidemic of HTN among South Asian children. In a recent comparison of office-based BP from seven countries, South Asian children had the highest and second-highest median diastolic and systolic BP measurements, respectively.^17-19^ Our previous work has identified that South Asian adults living in Canada have a higher prevalence of HTN; twice the prevalence of type 2 diabetes; higher body fat; and lower levels of physical activity compared to white Caucasians, all of which contribute to their higher CVD burden.^20-24^ Many of these cardiometabolic risk factors are also present in South Asian children, who are more likely to have excess adiposity compared to white Caucasian children.^25-27^

As the South Asian population grows in Canada, efforts to identify and mitigate CVD risk factors at an early stage are of critical importance. Thus, accurate identification of HTN by ABPM among South Asian children should be a priority. However, before ABPM can be applied in routine pediatric practice, it first needs to be validated with normative data representative of multi-ethnic populations. As an initial step to address this critical knowledge gap in research and clinical care, ASHA (Deriving Normative 24-hour Ambulatory Blood Pressure Monitoring Data Among SoutH Asian Children) study has been designed to establish normative ABPM data among a large cohort of South Asian children living in 2 of Canada’s most populated provinces, Ontario, and British Columbia (BC).

## Study Objectives

### Primary Objective

Establish normative height-sex and age-sex-specific reference values for 24-h ABPM measurements among non-overweight South Asian children and adolescents (aged 5-17 years) living in Ontario and BC.

### Secondary Objectives

1. Determine the extent to which various ABPM parameters differ across body mass index (BMI) classifications (normal *vs.* overweight *vs.* obese)
2. Determine the extent to which South Asian normative ABPM data differ from existing white Caucasian (German) and East Asian (Hong Kong) normative data
3. Evaluate the association between children’s habitual movement behaviors (physical activity, sedentary time, and sleep) and ABPM parameters
4. Evaluate the association between diet quality and ABPM parameters, using a simple diet score accounting for major dietary contributors to pediatric BP

### Hypothesis

The overarching goal is to improve cardiovascular health of South Asian children in North America and HTN is an *immediately targetable* risk factor. For this reason, accurate measurement of BP by ABPM among South Asians is critical. Hence, the primary objective is not directly hypothesis-driven but aimed at generating the ***required*** 24-h ABPM normative data. The secondary objectives are hypothesis-driven and aimed at internally validating this ABPM normative data.

The working hypothesis is that overweight or obese South Asian children will have higher ABPM measures than normal weight individuals; South Asian children will have higher BP compared to white Caucasians and East Asians; children with optimal movement behaviors (ie, high physical activity, low sedentary time, adequate sleep) and those with an optimal diet (ie, high intake of fruits and vegetables, dairy, and whole grains and low intake of sugary drinks, processed salty foods, soft drinks, and sweets) will demonstrate healthier ABPM profiles.

## Methods

### Study Design

This is a cross-sectional study with a quasi-representative sample of South Asian children living in Southern Ontario (Greater Toronto and Hamilton area, including the Peel Region) and Greater Vancouver, British Columbia (including Surrey). Hamilton Integrated Research Ethics Board approval for this study has been obtained.

### Eligibility Criteria

Children (aged 5-17 years) will be included if they have at least three South Asian grandparents.^28,29^ This age cut off was chosen because ABPM is recommended for children ≥5 years old. Children with pre-existing medical conditions (Table 1) or using specific medications known to affect BP will be excluded.

**Table 1.** Inclusion and Exclusion Criteria for the ASHA Study.

Inclusion criteria:
1. Aged 5-17 years at the time of the study enrollment 2. South Asian descent (defined as having at least 3 of 4 grandparents who had ancestral origins in India, Pakistan, Bangladesh, Sri Lanka, or Nepal).
Exclusion criteria:
1. Inability or unwillingness to provide informed or surrogate consent 2. History of previously diagnosed hypertension from a healthcare provider or previous treatment with lifestyle measures or antihypertensives (we will not exclude children with previously elevated BP readings that have not been diagnosed with hypertension and/or have not had any recommendations for lifestyle of medication management) 3. History of cardiovascular, renal, or endocrine disorders - Cardiovascular: aortic coarctation, any congenital heart disease, and cardiomyopathy - Renal: renal artery stenosis/abnormalities, renal vein thrombosis, acute glomerulonephritis, nephrotic syndrome, long-term kidney disease, end-stage renal disease (dialysis or transplantation), polycystic kidney disease, congenital anomalies of the kidneys and urinary tract (CAKUT) - Endocrine: thyroid disorders, congenital adrenal hyperplasia, Cushing syndrome, hyperaldosteronism, hyperparathyroidism, hypercalcemia, diabetes mellitus (type 1 or 2), diabetes insipidus, and pheochromocytoma - Other: malignancy, systemic lupus erythematosus, systemic juvenile idiopathic arthritis, Kawasaki disease, neurofibromatosis, tuberous sclerosis, and fibromuscular dysplasia 4. History of clinically relevant inborn errors of metabolism or pathological genetic disorders 5. Current use of medications known to affect BP, regardless of dose/duration or indication for use (defined as any ACE inhibitor, angiotensin-II receptor blocker, calcium channel blocker, thiazide-like diuretic, potassium-sparing diuretic, alpha-blocker, or beta-blocker) 6. Current use of oral/intravenous steroids and/or ≥1 month of cumulative oral/intravenous steroid use in the past 12 months regardless of dose (includes prednisone, prednisolone, dexamethasone, and hydrocortisone). This does not include inhaled, intranasal, ophthalmic, or topical corticosteroids. 7. Current use of any stimulant medication (includes amphetamine/dextroamphetamine, dextroamphetamine) 8. Actively pregnant or breastfeeding

*Note.* ASHA = South Asian cHildren in Canada; BP = blood pressure; ACE = Angiotensin-converting enzyme.

For our primary objective, only nonoverweight children will be included, consistent with 2017 American Academy of Pediatrics (AAP) HTN guidelines.^30^ For the secondary objectives, additional overweight/obese children will be enrolled for comparison with nonoverweight children.

### Participant Recruitment

A comprehensive list of local schools, places of worship (ie, temples, mosques, and churches), and community centers within the Greater Toronto and Hamilton area and Greater Vancouver will be compiled. All institutions will be invited to participate. After approval from school boards, invitation packages will be distributed (either in person or electronically) to the parents/guardians of all students enrolled at participating schools. These packages will include an invitation letter, study description, and required consent forms. Study information will also be disseminated in the newsletters of participating schools, and information stands will be set up at school events to advertise the study to parents/guardians.

After obtaining consent, study information leaflets that are accessible in multiple languages (English, Hindi, Punjabi, Gujrati, and Urdu) will be distributed. In addition, with the required permission, research staff will be made available at booths during health fairs and public events, distributing study packages with the pertinent contact information. Individuals that are interested in participating will be able to communicate with a research coordinator (via phone or email) to discuss the study and arrange an appointment to be fitted for the ABPM and accelerometer devices. Participant health card numbers will be recorded for future linkage with Ontario administrative databases housed at ICES.

### Questionnaire and data management

Parents/guardians (for children <12 years) and adolescents (≥12 years) will be asked to complete questionnaires on covariates (including age/sex), physical activity, sedentary and sleep behaviors. Children ≥12 years will be asked confidentially about smoking, vaping, and alcohol use.^31^ Children’s diet quality (reflecting intake of fruits and vegetables, dairy, whole grains; and processed foods, salty snacks, sugary drinks, and sweets) will also be assessed, adapting a *diet quality score* that has previously been published by the research team.^32^ Briefly, diet quality will be scored as follows: 1 point is given for consuming more than the study population median of green vegetables, fruits, whole grains, and so on.; or less than the study population median of sugary snacks, sweets, and so on. The final score ranges from 0 to 6; diet quality is classified as low (0 or 1), intermediate (2 or 3), or high.^4-6^ Information on annual household income, parenteral employment, and marital status will also be collected to derive a validated *social disadvantage index*; which combines social and economic factors into a single measure and has previously been published by the team.^33^ The social disadvantage score ranges from 0 to 5, with 5 representing the greatest social disadvantage. Among children ≥12 years, we will also use a validated *perceived stress scale* (PSS-10)^34^ to measure perception of psychological stress, which can also affect BP.^35^ Score ranges from 0 to 40, and higher scores correspond to a higher level of perceived stress.

Study questionnaires will be completed by parents/guardians electronically using REDCap Surveys. Participants who are unable to complete the electronic survey will complete paper questionnaires, that will be entered by research coordinators into a secure REDCap database. Ambulatory BP monitoring software outputs and movement behavior data for each participant will also be entered into REDCap and will be linked using unique identifiers to these questionnaires and forms. REDCap databases will be housed on a secure centralized server at McMaster University.

### Anthropometric Measurements

A team of trained research staff will collect anthropometric data at the ABPM fitting visit. Standing height and body weight will be measured. There is no consensus on which anthropometric method is best to define overweight/obesity among children. Each of the commonly used indicators of childhood obesity (ie, body mass index [BMI], waist and hip circumference, waist-to-height ratio, and body fat percentage) are associated with cardiometabolic risk.^36,37^ Moreover, this research group has previously shown that all of these adiposity indicators are strongly associated with HTN among South Asian children.^22^ Hence, overweight or obesity in the study population will be defined if *any of the following criteria are met: BMI*: Overweight if BMI >85th percentile, obesity as BMI >97th percentile, using World Health Organization (WHO) reference data.^38^ *Waist circumference*: Obesity will be classified as sex-specific waist circumference >90th percentile. *Waist-to-height ratio (WHtR)*: calculated as (waist circumference [cm]/height [cm]) and transformed into WHtR *z*-score; obesity defined as a ratio of >0.5. *Body fat percentage*: estimated by bioelectrical impedance analysis (BIA); Overweight if body fat >85th percentile, obesity if >95th percentile based on existing normative data.^39^ Casual BP will be measured from right arm by auscultatory method as per AAP 2017 guidelines.^30^ Three readings, at least 3 minutes apart, will be taken and averaged. Participants will be asked to perform the hand grip strength test using Takei Handgrip Dynamometer. Urine samples will be collected from participants for storage and future analysis for creatinine, sodium, and potassium.

### Ambulatory BP monitoring

All ABPM measurements will be taken from the nondominant arm. All measurements will be performed according to 2014 American Heart Association ABPM guidelines.^40^ The Spacelabs 90217 ABPM device has been validated for use in children and can measure heart rate, systolic and diastolic BP by oscillometric method.^41^ The validation study enrolled 112 American children aged 6 to 17 from diverse ethnic backgrounds, finding that the Spacelab 90217 ABPM device accurately measured BP, compared against a British Hypertension Society protocol. Ambulatory BP monitoring readings will be obtained every 30 min during the day and 30 min during the night for a 24-h period. All participants will be instructed to maintain their usual activity level but will be requested to avoid swimming or bathing for the 24-h period. Furthermore, participants and parents/guardians will be instructed to record bedtime and wake time in a diary, which will be used to categorize daytime and nighttime readings.

Ambulatory BP monitoring profiles with less than 50% of the proposed daily or nighttime readings will be excluded. Profiles where there was device disconnection for ≥1.5 consecutive hours will also be excluded. In these instances, with consent from parents/guardians, an ABPM device will be re-fitted, and data will be re-collected for 24-h. The initial recording will be excluded from the study. If, however, during the second data collection process, an adequate profile is not obtained, the subject will be excluded from the study. In instances where the individual is unable to tolerate the device resulting in early removal, the participant will not undergo data re-collection. In the event of device loss or damage, the participant will also not undergo data re-collection. If there is a significant discrepancy (≥3 h physical activity time) between reported physical activity (by questionnaire) and physical activity monitor measurements, the family will be contacted for clarification. If it is determined that the child’s physical activity during the measurement period was significantly different from normal, data re-collection will be offered, and the initial recording will be excluded from the study.

### ABPM Parameters

The following ABPM parameters will be measured: mean systolic BP (mmHg), mean diastolic BP (mmHg), mean arterial pressure (mmHg), systolic and diastolic BP load (percentage of abnormal readings; >95th percentile), and nocturnal dipping (percentage of BP reduction while sleeping compared to daytime; normal >10%). Except for nocturnal dipping, these outcomes will be quantified for 3 time periods: overall 24-h, daytime, and night-time periods.

### Movement Behaviour Assessment

During the 24-h ABPM period, participant activity levels will simultaneously be tracked using a wearable physical activity monitoring device. Accelerometry is an objective method of assessing free-living activity and has become the method of choice for the measurement of physical activity, sedentary, and sleep time in children.^42^ We will use the ActiGraph GT3X-BT accelerometer, which is a small, unobtrusive device validated for pediatric use.^43-46^ The ActiGraph and ABPM devices will be time-synced and worn over the same 24-h period. All participants will be instructed to maintain their usual activity levels during this 24-h recording period. Physical activity data will be retrieved from the devices and stored together with ABPM data. If the activity monitor recording is inadequate (due to device disconnection, battery failure, etc.), the recording will be excluded but the participants ABPM data will still be included in the study.

To accurately estimate habitual movement behaviors, participants will then continue wearing the ActiGraph device 24-h per day (with the exception of water activities) for a total of 7 consecutive days, as recommended by Trost et al.^47^ Participants will be asked to record any device removals, wake and sleep times. Accelerometers will record accelerations at a sampling frequency of 30 Hz, which will then be filtered and converted using a proprietary algorithm into activity counts in 3-s epochs (sampling intervals) to capture the typical “stop-and-go” activity patterns observed in children.^48^ Only participants with ≥4 days with ≥10 h of average wear time per day will be included, which has been shown to provide a highly reliable (~92%) estimate of movement behaviors.^49^ Using established Evenson cut-points,^43^ the daily minutes of sedentary time (defined as time spent with activity counts ≤25), all physical activity (time spent with activity counts >25), light-intensity activity (time spent with activity counts between 26-573), and moderate-to-vigorous physical activity (time spent with activity counts ≥574) will be calculated, which is the focus of public health recommendations.^50^

Total sleep time will be quantified using the refined Tudor-Locke algorithm, which has been validated for use in children.^51^ This 2-step process first uses the Sadeh sleep-wake algorithm to establish the probability of sleep, where probability of sleep for a given epoch = 7.601 − (0.065 × mean activity counts for 5 min before the epoch) − ln(1.08) − (0.056 × standard deviation activity counts for 6 minutes after the epoch) − 0.703 × ln (activity count of the epoch). If the probability of sleep is ≥0, then the given epoch is scored as sleep; if the probability of sleep is <0, the epoch is scored as awake.^52^ This algorithm has been validated using polysomnography data. Next, all epochs scored as sleep are verified against accelerometer-based measures of relative position. When probability of sleep and relative position both indicate sleep, the epoch will be assigned a final label of sleep.^53^ Sleep epochs for each day of accelerometer wear will be summed, and reported as average time spent sleeping per day, in minutes.

### Safety Monitoring

Ambulatory BP monitoring is a relatively noninvasive tool; however, in the event, a participant experiences intolerance to the device (eg, due to discomfort), parents will be advised to remove the BP cuff for a period of up to 1 h. If they are unable to re-fit the cuff after this time and/or the participant remains intolerant to the device, it will be recommended that they disconnect and return the device.

All participants/families will be notified of the BP classification of their clinic BP readings (as per AAP guidelines) during their final study visit (24-h after the initial readings). They will be provided a handout documenting their AAP BP classification and recommended timing of BP re-measurement. They will be asked to contact their primary-care physician for follow-up of abnormal readings, if necessary. If the participant has evidence of hypertensive urgency or emergency (ie, symptomatic HTN, BP >30mmHg above the 95th percentile, or >150/90mmHg in an adolescent) during their “office-based” readings, they will not proceed with ABPM monitoring, and it will recommend that they seek immediate care in an emergency department setting. If the ABPM reading results in a more severe BP classification than previously recorded during “office-based” readings, the family will be contacted to notify them of this difference and to update the recommendations made regarding BP and medical follow-up.

### Study Procedure and Timelines

**Figure 1.**
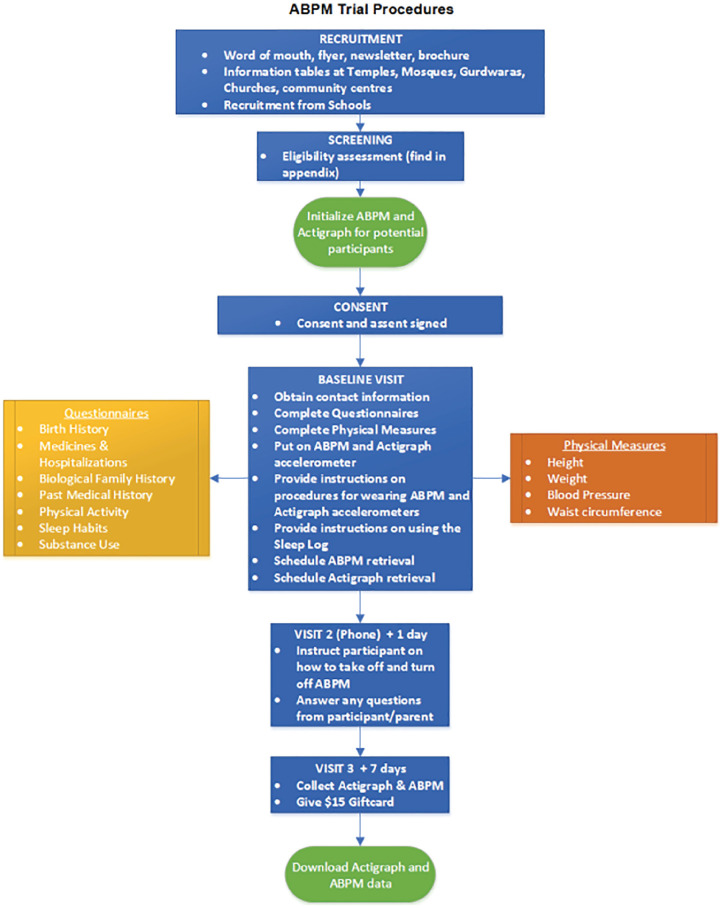
Participant flow diagram. *Note.* ABPM = ambulatory blood pressure monitoring.

All participants will have a baseline visit (visit 1) which will include completing questionnaires (electronically or paper), collecting anthropometric data, providing instructions on ABPM and ActiGraph, and attachment of both devices. The research coordinator will call the family 24-h later (visit 2) to instruct them to take-off the ABPM device and to answer any questions they may have. The final visit (visit 3) will be in-person on day 8, where ABPM and ActiGraph devices will be returned (Figure 1). The aim is to complete recruitment within 4 years, followed by knowledge translation activities. Based on our assessment of the eligible population size, established support from local recruitment sites (eg, school boards, places of worship, and community centers), our study team’s past experience recruiting within this population, and the resources available for recruitment, we believe that target recruitment is feasible within this timeframe.

### Sample Size Calculation

Height-sex specific ABPM curves ranging from 120 to 185 cm for boys and to 175 cm for girls, at intervals of 5 cm will be created. Assuming a standard deviation (SD) in mean systolic BP of 8 mmHg by ABPM^54^ and a desired precision of measurement of ±2.5 mmHg (95% CI), 70 nonoverweight participants within each height and sex group (a total of 980 boys between 120-185 cm and 840 girls between 120-175 cm) will be required for acceptable precision (SAS software, version 9.4; SAS Institute; Cary, NC). Oversampling will be required to account for 15% loss of participants due to inadequate ABPM recordings (machine malfunction, accidental cuff removal, etc.). This estimate is based on reported frequencies of inadequate pediatric ABPM readings: 8% of children (German study), 11% (Hong Kong study), and 13% to 17% (other pediatric cohorts).^55-59^ Therefore, a total of 2113 nonoverweight participants are required for the primary objective.

This study is also adequately powered for age-sex-specific ABPM curves. Keeping the same assumptions as above, a sample size of 1932 children will be required, which includes 70 normal weight participants within each age (5-17 years) and sex group and 15% data loss. This sample size is also consistent with recommendations to include at least 50 to 100 individuals in each LMS analysis group, and with previous German (1141 participants) and Hong Kong (1445 participants) studies.

According to Public Health Agency of Canada, around 30% Canadian children are overweight or have obesity.^60^ Hence, we will enroll 633 additional children to have adequate power for the secondary objective. Therefore, the total sample size for the study is 2746 (2113 + 633) children.

### Statistical Analysis for the Primary Objective

Among nonoverweight children, height-sex, and age-sex-specific centile curves (5th, 50th, 75th, 90th and 95th) for mean systolic BP, mean diastolic BP, and mean arterial pressure will be created. Based on the findings from the German and Hong Kong studies, it is expected that the distributions will be skewed. For this reason, the LMS method will be applied.^61^ This approach allows the incorporation of any degree and direction of skewness in the distributions. First, to normalize the skewed distribution data, the Box-Cox power transformation will be applied for each height-sex-specific level. By incorporating this Box-Cox power curve (L), a smoothed median curve (M), and a smoothed coefficient of variation curve (S) of the distribution, height-sex-specific percentile curves will be created. This analysis will be performed using LMS Program (version 12.43; Institute of Child Health, London, UK). The LMS method was also used in previous German and Hong Kong studies, which will allow for easier comparisons between cohorts.^54,56^ This method will be repeated to determine age-sex-specific centiles. These analyses will be performed for all three time periods (overall 24-h, daytime, and nighttime periods). All statistical tests will be 2-sided, with a *P* value < .05 considered to be statistically significant.

### Statistical Analysis for Secondary Objectives

All secondary objective analyses will be performed for the three time periods: overall 24-h, daytime, and nighttime periods.

#### To determine mean differences in ABPM measures across BMI classification

Multivariable regression analysis adjusting for age and other covariates (diet quality score, social disadvantage index, perceived stress scores) will be performed. To investigate potential interactions between obesity and sex, sex will individually be added into the model alongside an interaction term. The interaction *P* value will determine if there is a significant difference in ABPM measures among overweight/obese children and sex.

#### To descriptively compare our normative data against the German and Hong Kong cohorts

We will plot the height-sex and age-sex-specific 5th, 50th, and 95th centile curves of each cohort alongside each other for three ABPM parameters: mean systolic BP, mean diastolic BP, and mean arterial pressure. Mann Whitney *U*-test will be used to determine differences between our data with each of the other 2 cohorts (Germany and Hong Kong).

#### To evaluate the association between child’s habitual movement behaviors and ABPM measures

Compositional data analysis will be used, as outlined by Chastin et al.^62^ The advantage of this approach is that it accounts for the fact that physical activity, sedentary time, and sleep time are interconnected behaviors that make up a whole day (ie, sedentary time + physical activity + sleep = 24-h day). The specific habitual movement behaviors of interest include average daily sedentary time (minutes per day), time spent in light-intensity physical activity, moderate-to-vigorous intensity physical activity and sleep, all measured by accelerometry. Briefly, the geometric mean of movement behaviors will first be calculated, which will provide an estimate of the proportion of time spent in each movement behavior per day—the key exposure variables (Table 2). Next, the dispersion of the compositions will be estimated using a variation matrix.^62^ The composition, which is summarized by the geometric mean and its variation matrix, will be transformed using the isometric log-ratio transformation and incorporated into regression analyses.

**Table 2.** Key Variables on Which Data Will Be Collected Through Validated Questionaries.

Variable	Parent	Child
Ethnicity	X	
Country of Origin (India, Sri Lanka, Pakistan, Nepal, and Bangladesh)	X	
Education	X	
Employment	X	
Income	X	
Family history of CVD, diabetes, HTN	X	
Past medical history		x
Sleep time^a^		x
Physical activity^a^		x
Office BP (auscultatory method)		x
Diet quality score (consumption of fruits and vegetables, processed foods/salty snacks, low-fat dairy, whole grains, sugary drinks, and sweets)	X	x
For children 13 years and older: smoking, vaping, alcohol For children 12 years and older: perceived stress score		x
Height, weight, waist and hip circumference, body fat, hand grip strength, urine sample		x
Age and sex		x

*Note.* CVD = cardiovascular disease; HTN = hypertension; BP = blood pressure.

a The questionnaire data for these variables will be used for quality control purposes.

Regression models will be used to estimate:

1. The independent contribution of each movement behavior to ABPM outcomes (eg, what is the relationship between sedentary time and ABPM parameters?)
2. The combined effect of the composition of movement behaviors on ABPM outcomes, adjusted for age and BMI (eg, how do the collective movement behaviors predict ABPM parameters?)
3. The effect of displacing time from one behavior into another on ABPM outcomes, adjusted for age BMI (eg, what is the effect of replacing 30 min of sedentary time with sleep on ABPM parameters?)

#### To evaluate the association between child’s diet quality and ABPM measures

Univariate and multivariable regression analyses will be used to determine the association of the diet quality score (categorized as low, intermediate, and high) with the ABPM parameters after adjusting for other covariates.

## Discussion

The study results will have an immediate impact, providing normative reference values for ABPM interpretation among South Asian children living in Canada. This will facilitate more accurate HTN diagnosis and monitoring among South Asian children, potentially leading to improvements in their cardiometabolic health. This study will seek to overcome several limitations of the existing normative data which was primarily established in white Caucasian children.

No previous studies have examined the relationship between BP and combined movement behaviors in South Asian children. Other modifiable risk factors for pediatric HTN are obesity, high salt intake, and low fruit/vegetable consumption.^63,64^ This study will give a better understanding of the relationship between ABPM and important modifiable lifestyle factors, identifying suitable intervention targets for at-risk and hypertensive South Asian children.

Undertaking a large multicenter Canadian study does pose significant challenges with regards to successful recruitment, as adequate patient enrollment is integral to the applicability of our findings. Based on 2016 census data, there are 87,770 South Asian children (5-19 years) in the Peel Region and 42,615 in the Vancouver central metropolitan area. The aim is to recruit 2746 children over 5 years, which amounts to 2% of this population, to achieve adequate sample size. Previous studies on this scale have shown us that random sampling is not feasible for this population.^22,28,29^ Instead, convenience sampling will be used. By obtaining approval from local school boards, a large student population will be more easily accessible, allowing for direct dissemination of information with prospective participants and their parents/guardians. In the Peel Region specifically, for another project, the study team were able to recruit approximately 32% of contacted participants and of those, over 99% were able to complete the study.^29^ Similar recruitment rates are expected, using similar strategies as those outlined above. Sample representativeness will also be evaluated by determining the distribution of South Asian ethnic subgroups and comparing these against local 2016 census data (information will be collected on the country of origin, employment status, income, and education level) after recruitment of every 200 children.

In addition to ensuring high levels of enrollment, the potential selection bias must be addressed, which is introduced with an opt-in recruitment strategy. This will primarily be avoided by increasing the accessibility of study procedures and by performing in-person visits in convenient settings (ie, at home or school). In addition, selection bias will also be mitigated in the following ways: by inviting all eligible schools, places of worship, and community centers to participate; by contacting all parents/guardians of South Asian children at included schools; by providing study information in multiple languages to ensure accessibility and providing telephone interpretation services for study visits as required; and by providing flexible hours for study visits to accommodate schedules.

When running a large, multicenter study, it is imperative that data collection is standardized to allow for comparisons to be made between sites. To do this, all the same data gathering techniques (eg, office BP measurement) and type of equipment (ABPM and activity monitoring devices) will be used. Standardized training for research staff in all locations will be implemented. The safe storage and transfer of data is integral to this project, and a centralized secure database with required technological support will be used to ensure security of all data collected.

This study relies heavily on the use of ABPM and activity monitoring devices. Therefore, it is vital that these devices function correctly and that the team is equipped to troubleshoot problems. The research team will be trained specifically with the models used in this study and will be well equipped to troubleshoot common issues. Participants/caretakers will be given the contact information of research staff who they can contact in the event they run into technical issues during the data collection period. There may be participants who are not able to tolerate the devices and those who experience device failure or accidental removal, resulting in incomplete ABPM profiles. For these reasons, more participants than are statistically required will be enrolled, accounting for previously reported rates of participant drop-out or incomplete ABPM measures. In addition, specific procedures for addressing the problem of incomplete profiles have been developed. As outlined previously, profiles with short device disconnection periods (<1.5 h) will be accepted. For instances of prolonged disconnection, a second 24-h data gathering period will be offered, depending on the reason for device disconnection. To ensure that the activity data gathered by questionnaire is comparable to values obtained with activity monitoring devices, up to a 3-h discrepancy will be allowed. Activity guidance will also be provided to the family at the in-person ABPM fitting visit via a written handout, to attempt to minimize the likelihood of significant discrepancies in patient activity. It is also expected that younger participants may experience decreased tolerance for ABPM devices as they may be unaware of when to expect the readings. However, these ABPM devices and accelerometers have previously been used successfully in pediatric cohorts, including young children.^41,43-46^

To maximize the feasibility of this study, bloodwork was decided not to be included, nor the recruitment of a non-South Asian comparator cohort. However, the results from this study will form the basis of a research platform to address future questions, including metabolomic and genomic profiles related to BP classification, with the anticipated future collection of additional urine and DNA samples.

The multidisciplinary team will launch several knowledge translation and advocacy projects based on our findings. To guide relevant integrated knowledge translation processes from study inception to data dissemination and evaluation, the Knowledge-to-Action (KTA) framework will be used.^65^ To support best practices around relevant and impactful recruitment, data collection, and data dissemination activities, a diverse Research Advisory Committee (RAC) will be assembled with representatives from: youth, parents, teachers/educators, places of worship, city community centers, family physicians, Canadian Association of Pediatric Nephrologists (CAPN), Hypertension Canada, Canadian Pediatric Society (CPS), and Peel Region Public Health. The study’s results are most relevant to family physicians, general pediatricians, pediatric nephrologists, and cardiologists who care for children with HTN. An integrated KTA approach includes both knowledge creation and application. The primary goal of these initiatives will be to make this normative data widely accessible, easy to use, and well-integrated with available resources, in order to increase ABPM utilization among South Asian children and facilitate interpretation of this ABPM data with our reference values. To achieve this, we intend to develop a range of clinical tools and provide these tools at no cost on an open-access website. These tools will include ABPM reference value tables and centile curves, as well as a pocket reference guide with a simplified schematic for ABPM interpretation and classification. We will also design an online calculator where clinicians can input ABPM measures and generate an automated ABPM report, with classification based on our normative data. We plan to design a free mobile application featuring this calculator, for easy access in a clinic environment. We aim to include normative ABPM values from other pediatric populations (eg, Caucasian European, East Asian, and future ethnic group data) into this calculator, with potential integration with commonly used electronic medical record platforms.

The platforms of Canadian Association of Pediatric Nephrologists, Hypertension Canada, International Pediatric Nephrology Association, International Society of Nephrology, and Canadian Society of Nephrology will be used to widely disseminate our findings and advocate for the use of ethnic-specific ABPM normative data in South Asian children. We will develop short videos and infographics supplemented with educational resources on lifestyle measures to mitigate HTN and CVD. These will also be distributed to participating schools, places of worship, and community centers. Partnerships with regional and national public health agencies will allow us further distribute results to community-based primary-care providers.

To increase the generalizability of our results on an international level, data will subsequently be compared to ABPM normative data that is being simultaneously collected in India by a collaborative research team. This will provide insight into the impact of geographical location and environmental factors on ABPM measures among individuals of similar ethnic background. Normative data from these 2 populations could be subsequently combined to create a “global” pediatric South Asian normative dataset. Furthermore, this study will serve as a model for determining normative ABPM data in other pediatric populations, allowing for additional normative standards to be developed for clinical use.

## Conclusions

Ambulatory BP monitoring is a useful tool for the diagnosis and management of childhood HTN. Establishing normative ABPM reference data for South Asian children may increase its clinical utilization in this population, resulting in more accurate HTN assessment and promoting improved BP control. Comparison against existing German and Hong Kong data will provide information on geographic, ethnic, and temporal changes in childhood BP. Furthermore, this study will serve as a model for determining normative ABPM data and allow for comprehensive standards to be developed for other pediatric populations.
